# A Distinct, Non-Virion Plant Virus Movement Protein Encoded by a Crinivirus Essential for Systemic Infection

**DOI:** 10.1128/mBio.02230-18

**Published:** 2018-11-20

**Authors:** Wenjie Qiao, Vicente Medina, Yen-Wen Kuo, Bryce W. Falk

**Affiliations:** a Department of Plant Pathology, University of California, Davis, California, USA; b Department of Crop and Forest Sciences, University of Lleida, Lleida, Spain; University of Nebraska—Lincoln; University of Florida; State University of New York

**Keywords:** *Crinivirus*, *Lettuce infectious yellows virus*, P26, movement protein, systemic infection

## Abstract

Plant-infecting viruses utilize various strategies involving multiple viral and host factors to achieve successful systemic infections of their compatible hosts. *Lettuce infectious yellows virus* (LIYV), genus *Crinivirus*, family *Closteroviridae*, has long, filamentous flexuous virions and causes phloem-limited infections in its plant hosts. The LIYV-encoded P26 is a distinct non-virion protein that shows no similarities to proteins in current databases: it induces plasmalemma deposits over plasmadesmata (PD) pit fields and is speculated to have roles in LIYV virion transport within infected plants. In this study, P26 was demonstrated to be a PD-localized protein, and its biological significance was tested *in planta* by mutagenesis analysis. An LIYV P26 knockout mutant (P26X) showed viral RNA replication and virion formation in inoculated leaves of Nicotiana benthamiana plants, but failed to give systemic infection. Confirmation by using a modified green fluorescent protein (GFP)-tagged LIYV P26X showed GFP accumulation only in infiltrated leaf tissues, while wild-type LIYV GFP readily spread systemically in the phloem. Attempts to rescue P26X by complementation in *trans* were negative. However a translocated LIYV P26 gene in the LIYV genome rescued systemic infection, but P26 orthologs from other criniviruses did not. Mutagenesis *in planta* assays showed that deletions in P26, as well as 2 of 11 specific alanine-scanning mutants, abolished the ability to systemically infect N. benthamiana.

## Abstract (precis)

Plant viruses encode specific proteins that facilitate their ability to establish multicellular/systemic infections in their host plants. Relatively little is known of the transport mechanisms for plant viruses whose infections are phloem limited, including those of the family *Closteroviridae.* These viruses have complex, long filamentous virions that spread through the phloem. *Lettuce infectious yellows virus* (LIYV) encodes a non-virion protein, P26, which forms plasmalemma deposits over plasmodesmata pit fields, and LIYV virions are consistently found attached to those deposits. Here we demonstrate that P26 is a unique movement protein required for LIYV systemic infection in plants. LIYV P26 shows no sequence similarities to other proteins, but other criniviruses encode P26 orthologs. However, these failed to complement movement of LIYV P26 mutants.

## INTRODUCTION

The establishment of systemic infections by viruses in a compatible host plant involves specific interactions between virus and host proteins. Plant viruses move from the initially infected cells to neighboring cells via plasmodesmata (PD), which are the plasma membrane-lined intercellular connections across the cell wall, and then achieve long-distance transport via the vascular system to distal parts of the plants (1). Virus-encoded proteins have evolved to achieve these functions through collaborative interactions with other viral and host factors. Intercellular movement within mesophyll tissues has been well studied for viruses of different taxa, and different viruses utilize different strategies. Viruses such as como- and nepoviruses encode a movement protein (MP) that modifies PDs extensively into MP-lined tubules to facilitate viral intercellular movement in the form of virions (2). Tobamoviruses, represented by *Tobacco mosaic virus* (TMV), encode a single dedicated MP that increases the size exclusion limit of PDs and mediates cell-to-cell transport of a complex of viral RNA and MP-associated viral replication complexes (VRCs) (3, 4). Potexviruses, which also move as viral ribonucleoprotein complexes (vRNPs), depend on the virion capsid protein in addition to three MPs encoded in overlapping open reading frames (ORFs) called the “triple gene block” (TGB) that function coordinately (5). Unlike all the viruses mentioned above, potyviruses have no dedicated MP(s) but involve several viral proteins that have multiple roles in the virus infection cycle, to move through PDs as virions, including the cylindrical inclusion protein (CI), CP, helper-component proteinase (HC-Pro), viral genome-linked protein (VPg), and P3N-PIPO (6). While much is known about intercellular movement of plant viruses in mesophyll tissues, in contrast, much less is known regarding how different viruses achieve systemic transport via the vascular tissues, in particular the phloem.

Criniviruses have linear, bipartite positive-sense single-stranded RNA (ssRNA) genomes and belong to the family *Closteroviridae.* Viruses in this family possess the largest and most complex genomes and virions of all positive-sense ssRNA viruses infecting plants (7), and their infections are mostly phloem limited in their plant hosts. Efforts have been made to study viral and host factors relevant to the phloem-specific movement of closteroviruses in their host plants, but largely with limited progress due to the challenges of studying these phloem-limited, non-mechanically transmissible viruses. Previous studies on *Beet yellows virus* (BYV; genus *Closterovirus*) have identified several proteins, including the non-virion protein, P6, and five virion proteins (CP, CPm, Hsp70h [heat shock protein 70 homlog], P64, and P20), that are required for cell-to-cell and/or long-distance movement. P6 localizes and interacts with the endoplasmic reticulum (ER), whereas Hsp70h interacts with the virions in an asymmetric manner and targets PD by trafficking along the actomyosin (8–10). BYV is somewhat atypical of closteroviruses as it can be mechanically transmitted in some host plants, while others cannot. *Citrus tristeza virus* (CTV), another closterovirus which is phloem limited, codes for the protein P33, which is required for virus transport in some plant hosts (11). Information to date suggests that viruses of the family *Closteroviridae* move within the plant as virions; however, additional movement requirements for viruses in the genus *Crinivirus* have not been determined.

*Lettuce infectious yellows virus* (LIYV) is a well-studied crinivirus, and mutational analysis has so far shown that the virion proteins CP, Hsp70h, P59, and CPm are required for its systemic infection in plants, but a partial deletion of CPm still allowed systemic infection but disrupted LIYV whitefly transmissibility (12). Knockout mutations of these proteins most likely affected the stability of virions, the transport form of LIYV in plants; therefore, these results are not surprising. When tested by transient expression assays in plants, none of the LIYV virion proteins showed PD localization similar to the BYV Hsp70h, and whether they act like those of BYV remains unclear (13). The LIYV non-virion protein P26 has been suggested to be a possible movement-associated protein. P26 is dispensable for LIYV replication in tobacco protoplasts and accumulates in cells to give a unique cytopathology: the conical electron-dense plasmalemma deposits (PLDs) (14–17). PLDs are usually found over PD pit fields between companion cells and phloem parenchyma or adjoining sieve elements (SEs) and are consistently associated with large numbers of LIYV virions that appear to be oriented perpendicular to the plasmalemma (PM) and PD (16, 18, 19). Furthermore, virus-like particles were also occasionally observed within PD under the PLDs (19). P26 expression in plants is sufficient to induce the formation of the PLDs in the absence of other LIYV proteins, and when expressed from the heterologous TMV vector, unlike LIYV virions, TMV virions were not associated with P26-aggregated PLDs, suggesting that specific interactions occur between LIYV virions and the P26-formed structures (16, 17). Taken together, these findings suggest that P26 and the PLDs may have roles in LIYV systemic movement within plants.

In this study, we used transient expression assays to demonstrate that LIYV P26 is a PD-associated protein and prone to self-aggregation. Using the LIYV infectious cDNA clones and the Agrobacterium tumefaciens-mediated delivery system (20), we demonstrated that P26 was essential for LIYV systemic infection in Nicotiana benthamiana plants. Moreover, to gain further insight into the function and molecular determinants of P26, partial truncation and alanine-scanning mutagenesis were applied to identify regions and amino acids that influence P26 function and viral systemic infection.

## RESULTS

### LIYV P26 is associated with plasmodesmata.

**FIG 1:**
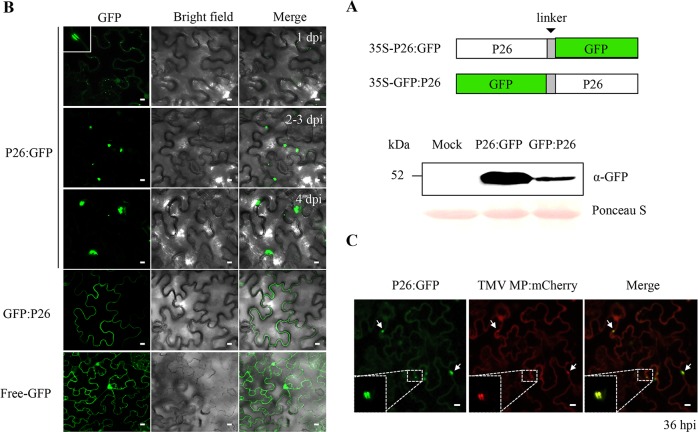
Subcellular localization of the transiently expressed P26 and GFP fusion proteins. (A) Schematic representation of the P26:GFP and GFP:P26 fusion constructs and protein expression confirmed by immunoblotting using anti-GFP antibody. “Mock” indicates the buffer-inoculated control. The Ponceau S-stained RuBisCO large subunit serves as a loading control. (B) Confocal imaging of P26:GFP- and GFP:P26-expressing mesophyll cells at 1 to 4 days postinfiltration (dpi) obtained under a 63× water immersion objective. The white square indicates one enlarged fluorescent dot of P26:GFP at 1 dpi, showing the characteristic plasmodesmata pit field. Free GFP was used as a control. (C) Confocal imaging of P26:GFP and TMV MP:mCherry-coinoculated mesophyll cells at 36 h postinfiltration (hpi). Arrows indicate some colocalized sites; one of them was enlarged, showing the characteristic plasmodesmata pit field. Scale bars, 10 μm.

Previous localization studies using immunofluorescence revealed that P26 accumulated as punctate spots near the cell periphery in Nicotiana tabacum protoplasts when expressed either from a heterologous virus, TMV, or from LIYV (17). To determine the LIYV P26-specific intracellular localization, N- and C-terminal green fluorescent protein (GFP) fusions with P26 (P26:GFP and GFP:P26) were transiently expressed in N. benthamiana leaves (Fig. 1A). The same distribution patterns were observed in the mesophyll cells of N. benthamiana leaves as those reported previously when P26 was expressed from TMV vector in N. tabacum protoplasts (17): P26:GFP showed small scattered punctae at 1 day postinfiltration (dpi) similar to the native LIYV-expressed P26 pattern, although probably due to protein overexpression and/or the self-interaction property of P26 (21), significant aggregation was observed later. In contrast, GFP:P26 was observed localized to the cell periphery throughout the plasmalemmas (Fig. 1B). Thus, both P26-GFP fusions targeted to the plamalemma, but P26:GFP appeared to accumulate in specific locations. In order to determine whether these punctate accumulations of P26:GFP were associated with PD, we compared their colocalization with mCherry-labeled TMV MP, one of the best-characterized viral MPs and which shows strong targeting to PD in plant cells (22, 23). The P26:GFP- and mCherry-labeled MPs colocalized, therefore, confirming the plasmodesmatal association of LIYV P26 (Fig. 1C).

### LIYV P26 is required for systemic infection in N. benthamiana plants.

**FIG 2:**
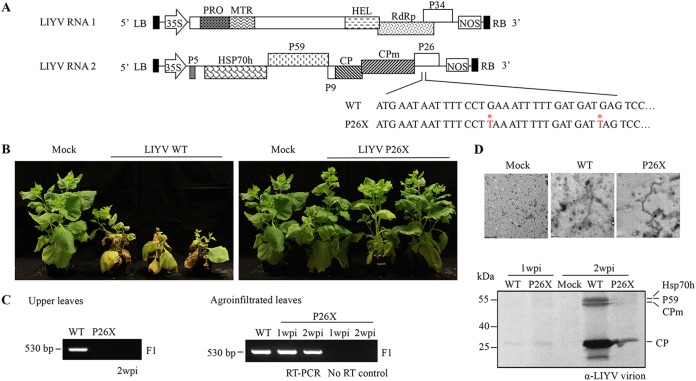
Impact of P26 on LIYV infection in Nicotiana benthamiana plants. (A) Schematic representation of the genomic organization of LIYV cDNA infectious clones. An LIYV P26 knockout mutant (P26X) was constructed by introducing two in-frame stop codons near the 5′ terminus of the P26 ORF (shown as DNA). The two nucleotides replaced were marked with asterisk (*) and are shown in red. LB, left border; RB, right border; 35S, 35S promoter; NOS, nopaline synthase terminator. (B) Phenotypes of LIYV WT- and P26X-agroinoculated N. benthamiana plants photographed at 4 weeks postinoculation (wpi). “Mock” indicates the buffer-inoculated control. (C) Detection of viral RNA accumulation by RT-PCR in upper noninoculated and agroinfiltrated leaves of LIYV WT- and P26X-agroinoculated plants at the time point indicated. The F1 primer set amplifying the sequence of LIYV CP (530 bp) was used. RNA samples derived from the agroinfiltrated leaves without RT were applied as a negative control. (D) Electron microscopy of partially purified virions from agroinoculated leaves. Virions extracted at 1 and 2 wpi were subjected to SDS-PAGE and immunoblotting using LIYV virion-specific antibody. The bands of LIYV virion proteins (Hsp70h, P59, CPm, and CP) are indicated on the right. Extraction from buffer-inoculated leaf tissues at 2 wpi (Mock) was used as a control.

To uncover the functional roles of P26 in viral infection in N. benthamiana plants, first an LIYV P26 knockout mutant (P26X) was generated by introducing two in-frame stop codons near the 5′ terminus of the P26 ORF in the LIYV wild-type (WT) infectious clone (Fig. 2A). The P26X mutant and wild-type LIYV were then separately introduced into leaves of N. benthamiana plants by agroinoculation. LIYV WT-infected plants showed typical interveinal yellowing symptoms at ca. 2 weeks postinoculation (wpi) and nearly died by 4 wpi, while the P26X mutant failed to induce any observable symptoms (Fig. 2B). The systemic accumulation of LIYV RNA was tested by reverse transcription-PCR (RT-PCR), using PCR primers flanking the LIYV CP coding region. LIYV RNA was only amplified from total RNAs extracted from the upper leaves of the LIYV WT-infected N. benthamiana plants, but not from the upper leaves of the LIYV P26X-inoculated plants (Fig. 2C). However, P26X replicated in the inoculated leaves as LIYV negative-sense RNA was detected by RT-PCR in both LIYV WT- and P26X-agroinoculated N. benthamiana leaves at 1 and 2 wpi; RNA samples without RT were used as negative controls (Fig. 2C). Conservation of the P26 mutations within the viral progeny was confirmed by sequencing.

Previous work showed that P26 knockout mutants replicated to similar levels as did wild-type LIYV in protoplasts (15). Here we purified and compared LIYV virions from both LIYV WT- and P26X-agroinoculated N. benthamiana leaves at 1 and 2 wpi and analyzed them by transmission electron microscopy (TEM) and immunoblotting with LIYV virion-specific antibodies (Fig. 2D). Virions showed similar morphology for both WT and P26X by TEM. Immunoblotting showed similar LIYV protein accumulation for the 1-wpi samples, but by 2 wpi, WT LIYV showed much greater accumulation, which is likely due to the ability of WT to spread and infect and spread to phloem cells. These results thus show that P26 is dispensable for LIYV replication and virion formation, but P26 is essential for systemic infection in N. benthamiana plants.

### Distribution of LIYV and the P26 mutant in N. benthamiana plants.

**FIG 3:**
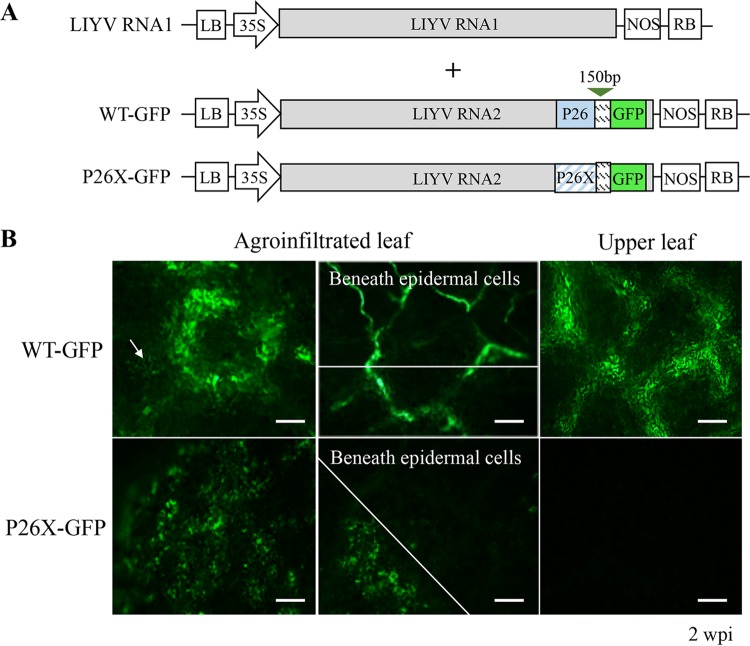
Distribution of the LIYV WT and P26X in agroinfiltrated Nicotiana benthamiana leaf tissues. (A) Schematic diagram of the genome organization of the GFP-tagged LIYV WT (WT-GFP) and P26X (P26X-GFP) cDNA infectious clones. A GFP ORF controlled by a 150-bp duplicated LIYV CP controller element (CE) was inserted between the P26 ORF and 3′-nontranslated region of LIYV RNA2. (B) The GFP fluorescence was monitored in the agroinfiltrated and upper leaves at 2 weeks postinfiltration (wpi) using a fluorescence microscope. The vascular system was observed by removing the epidermal layer from the lower side of the leaf tissue. An arrow indicates WT-GFP-infected epidermal cells showing fluorescence. Scale bars, 150 μm.

We next used GFP-tagged LIYV WT (24) and P26X to visualize their distribution in leaf tissues (Fig. 3A). The P26X-GFP construct was generated by introducing the same mutations of P26X into the WT-GFP construct, and GFP expression was monitored in the agroinoculated N. benthamiana leaves. Around 12 dpi, strong GFP fluorescence was only observed with a long-wavelength UV light primarily in the veins of leaves that were infiltrated by WT-GFP. Fluorescence microscopy was employed for further examination, both WT-GFP and P26X-GFP showed detectable GFP expression in epidermal cells of infiltrated leaves, while strong GFP signals were only generated from WT-GFP in adjacent cells and even vascular tissues. Since LIYV depends on the phloem for systemic infection, we looked closer into the vascular system by peeling off the epidermal layer of the infiltrated leaf tissues. No GFP signal was detected for P26X-GFP beneath the epidermal cells (Fig. 3B), suggesting that P26X-GFP replication was confined to the infiltrated cells and was unable to move and enter the vascular system for systemic infection. In contrast, the GFP expression for LIYV WT-GFP was obvious in vascular tissues and further tracked to other plant tissues, including stems, roots, and upper noninoculated leaves. Consistent with previous results, virus infection and GFP fluorescence were detected in WT-GFP-infected plant tissues and limited to the vascular system (24), while not in those inoculated by P26X-GFP (Fig. 3B; see Fig. S1 in the supplemental material). These results demonstrate that LIYV P26 likely functions in virus loading from initially infected cells into the phloem, but clearly, lack of P26 prevents LIYV systemic infection in N. benthamiana plants.

10.1128/mBio.02230-18.1 FIG S1 GFP expression in the stems and roots of N. benthamiana plants agroinoculated by WT-GFP and P26X-GFP at 4 weeks postinoculation. GFP fluorescence was visualized with a long-wavelength UV light. “Mock” indicates buffer-inoculated control. Dot blot analysis of the stem cross section was applied with anti-LIYV CP antibody. Download FIG S1, TIF file, 1.6 MB. Copyright © 2018 Qiao et al. 2018 Qiao et al. This content is distributed under the terms of the Creative Commons Attribution 4.0 International license.

### Systemic infection of P26X is rescued by a translocated P26 in the LIYV genome.

**FIG 4:**
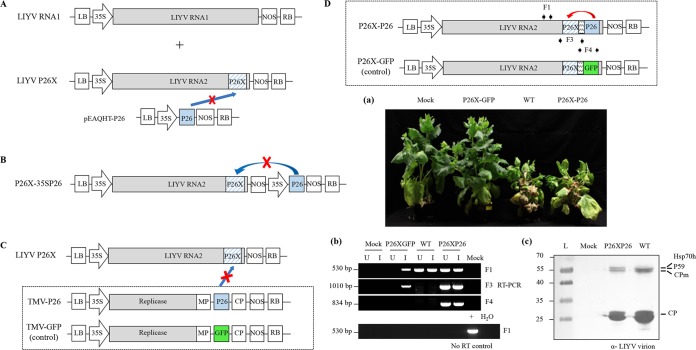
Strategies examined to rescue the systemic infection of LIYV P26X in Nicotiana benthamiana plants. Shown is failed complementation of P26X with (A) P26 expressed from binary vector pEAQHT (pEAQHT-P26), (B) P26 expressed from the P26 gene expression cassette removed from pEAQHT-P26 and introduced within the transfer DNA (T-DNA) borders adjacent to the expression cassette of P26X (P26X-35SP26), or (C) P26 expressed from a *Tobacco mosaic virus* (TMV) vector (TMV-P26). TMV expressing GFP (TMV-GFP) was used as a control. (D) Successful complementation of P26X with P26 expressed from a translocated P26 ORF in the LIYV genome (P26X-P26). P26X-GFP was used as a control. (a) Phenotypes of LIYV WT-, P26X-GFP-, and P26X-P26-infected N. benthamiana plants photographed at 4 wpi. “Mock” indicates the buffer-inoculated control. (b) Detection of viral RNA by RT-PCR with total RNA extracted from upper leaves (U) and agroinfiltrated leaves (I). F1, F3, and F4 primer sets were used to amplify the sequence of LIYV CP (530 bp), the original P26 ORF containing the P26 mutation (1,010 bp), and the added P26 ORF (834 bp). RNA samples derived from the agroinfiltrated leaves without RT were applied as a negative control. +, PCR-positive control with LIYV RNA2 cDNA plasmid; H_2_O, PCR-negative control without template. (c) Immunoblot analysis of LIYV virions extracted from the upper leaves of LIYV WT- and P26X-P26-agroinoculated plants.

As shown above, the LIYV P26 knockout led to localized infection. Therefore, to assess if the systemic infection could be restored by complementing P26 expression in *trans*, we used two approaches. First we constructed a binary plant expression vector, pEAQHT-P26, expressing P26 under the control of 35S promoter (Fig. 4A). A mixture of A. tumefaciens cultures (optical density at 600 nm [OD_600_] of 1.0) harboring the LIYV RNA1, LIYV RNA2 P26X (or LIYV RNA2 WT), and pEAQHT-P26 at a ratio of 1:1:1 was infiltrated into leaves N. benthamiana plants. The results showed that the P26X supplied in *trans* by pEAQHT-P26 was unable to complement and restore the ability to establish a systemic infection. Transient expression of P26 was confirmed in the infiltrated leaves by immunoblotting (see Fig. S2A in the supplemental material). However, we cannot be sure that P26 was expressed in cells also supporting P26X replication. Therefore, to ensure that P26 expression was in the same cells, the P26 gene expression cassette was excised from pEAQHT-P26 and introduced within the transfer DNA (T-DNA) borders adjacent to the expression cassette of LIYV RNA2 P26X to generate clone P26X-35SP26 (Fig. 4B). When A. tumefaciens cultures were then coinfiltrated with that of LIYV RNA1 at a 1:1 ratio (OD_600_ of 1.0), the P26X-35SP26 mutant again showed no systemic infection. Viral RNA was detected with LIYV WT systemically infected plants, but not with those inoculated by P26X-35SP26 mutant (Fig. S2B).

10.1128/mBio.02230-18.2 FIG S2 Validation of strategies used to rescue the systemic infection of LIYV P26X in Nicotiana benthamiana plants. (A) (Left) P26 expression from pEAQHT-P26 confirmed by immunoblotting using anti-P26 polyclonal antibody. (Right) Detection of viral RNA accumulation by RT-PCR with total RNA extracted from upper noninoculated leaves of LIYV P26X/pEAQHT-, LIYV P26X/pEAQHT-P26-, or LIYV WT/pEAQHT-coinoculated plants at 4 weeks postinoculation (wpi). (B) Detection of viral RNA accumulation in upper leaves of LIYV WT- and P26X-35SP26-agroinoculated plants at 4 wpi. An F1 primer set amplifying the sequence of LIYV CP (530 bp) was used for PCR. (C) (Left) GFP and P26 expression from a TMV vector confirmed by immunoblotting with anti-GFP and anti-P26 antibodies. (Right) Detection of TMV and LIYV RNA accumulation in upper noninoculated leaves of TMV-GFP/LIYV P26X- and TMV-P26/LIYV P26X-coinoculated plants at 4 wpi. TMV-IN and F1 primer sets were used to amplify TMV sequence flanking the insertions (930 bp) and the sequence of LIYV CP (530 bp). An RNA sample of LIYV WT-infected plant tissue was used as a control. (D) Subcellular fraction and immunoblot analysis of P26 protein expressed from LIYV WT- and P26X-P26-agroinoculated plants. LIYV CP was tested as a control. CW, cell wall; P1, 1,000 × *g* pellet; P30, 30,000 × *g* pellet; S30, 30,000 × *g* supernatant. The Ponceau S-stained RuBisCO large subunit serves as a loading control. Download FIG S2, TIF file, 0.8 MB. Copyright © 2018 Qiao et al. 2018 Qiao et al. This content is distributed under the terms of the Creative Commons Attribution 4.0 International license.

The above experiments would only allow for P26 accumulation in initially infiltrated cells. Therefore, to examine if continuously expressed P26 in *trans* could give recovery of the systemic infection of P26X, we utilized a GFP-labeled TMV vector, pJL24 (TMV-GFP), which is capable of systemic infection and expresses GFP systemically (25). The GFP ORF was replaced with the P26 ORF to generate TMV-P26. When TMV-P26 was agroinoculated to N. benthamiana plants, systemic infection resulted and P26 was detected in the upper leaves of agroinoculated N. benthamiana plants by immunoblotting (Fig. 4C). Coinfiltration experiments similar to those described above were then conducted to determine if TMV-P26 could complement LIYV P26X. A. tumefaciens cultures (OD_600_, 1.0) harboring the LIYV RNA1, LIYV RNA2 P26X (or LIYV RNA2 WT), and TMV-P26 (or TMV-GFP) were mixed at a ratio of 2:2:1 (OD_600_, 1.0) and infiltrated into leaves of N. benthamiana plants; however, systemic infection of LIYV P26X was not observed for any of the combinations (Fig. S2C). Taken together, these results show that P26 expression in *trans* was unable to rescue the movement-defective phenotypes of LIYV P26X.

We next replaced the GFP ORF of the LIYV P26X-GFP construct with the P26 ORF, thus, yielding LIYV RNA 2 containing a translocated P26 ORF: the resulting clones were designated P26X-P26 (Fig. 4D). When A. tumefaciens cultures containing LIYV RNA1 and P26X-P26 were coinfiltrated into leaves of N. benthamiana plants, typical symptoms comparable to those caused by the wild-type LIYV were seen, while the LIYV RNA1- and P26X-GFP-coinoculated controls gave no detectable symptoms (Fig. 4D). Viral RNA accumulation and integrity were examined in both agroinfiltrated and upper noninoculated leaf tissues by RT-PCR with LIYV negative-sense RNA-specific primers. The LIYV CP sequence was amplified from both WT- and P26X-P26-inoculated and upper noninoculated leaf tissues, but only in the inoculated leaves for P26X-GFP. The integrity of the original P26 ORF containing the knockout mutations and the translocated P26 ORF of P26X-P26 was confirmed by RT-PCR and sequencing. RNA samples without RT were used as controls to ensure that RT-PCR products resulted from RNA (Fig. 4D). Furthermore, LIYV virions were extracted from upper leaves of P26X-P26-infected plants and detected by immunoblot analysis with LIYV virion-specific antibody (Fig. 4D). P26 protein expressed from the translocated ORF was characterized by subcellular fraction and immunoblotting; the same distribution pattern as the wild-type LIYV expressed P26 was observed (Fig. S2D). Thus, the combined data showed that only a translocated P26 ORF in the LIYV genome could complement the function of P26, indicating that the expression location, quantity, and/or timing of the P26 protein in LIYV-infected cells might be important for its function.

### The functions of LIYV P26 cannot be complemented by its orthologs.

All viruses in the genus *Crinivirus* have similarly positioned RNA2 ORFs encoding for proteins similar in size to P26. However, the nucleotide and deduced amino acid sequences of these orthologs show little to no similarity, but they are predicted to have similar secondary structures (21). The P26 proteins encoded by orthologous genes from other criniviruses, including *Beet pseudoyellows virus* (BPYV), *Cucurbit yellow stunting disorder virus* (CYSDV), *Lettuce chlorosis virus* (LCV), and *Tomato chlorosis virus* (ToCV) were previously tested by yeast-two-hybrid (Y2H) assays, and all exhibited strong self-interactions similar to what is seen for LIYV P26 (21). Biochemical fractionation data also indicated some similar properties among LIYV, BPYV, and CYSDV P26 orthologs, although neither of the others causes the PLDs’ cytopathology (17). We wondered whether the orthologs are exchangeable for proper function in LIYV. Therefore, we replaced the P26 ORF in LIYV RNA2 with orthologous genes from BPYV, CYSDV, LCV, and ToCV. Agroinfiltration experiments showed that none could complement LIYV to give systemic infection in N. benthamiana plants (see Fig. S3 in the supplemental material). The results indicate that LIYV P26 is required for successful LIYV infection, and complementation is not achieved by orthologs from viruses of the same genus.

10.1128/mBio.02230-18.3 FIG S3 Substitution of *Lettuce infectious yellows virus* (LIYV) P26 with its orthologs. (A) Schematic representation of the genomic organization of LIYV cDNA infectious clones, of which the P26 ORF was replaced with its orthologous genes from *Beet pseudoyellows virus* (BPYV), *Cucurbit yellow stunting disorder virus* (CYSDV), *Lettuce chlorosis virus* (LCV), and *Tomato chlorosis virus* (ToCV). (B) Detection of viral RNA accumulation by RT-PCR with total RNA extracted from upper noninoculated leaves of Nicotiana benthamiana plants agroinoculated with these chimeric LIYV viruses (containing P26 orthologous genes). An F1 primer set amplifying the sequence of LIYV CP (530 bp) was used for PCR. Download FIG S3, TIF file, 0.2 MB. Copyright © 2018 Qiao et al. 2018 Qiao et al. This content is distributed under the terms of the Creative Commons Attribution 4.0 International license.

### Structural regions or amino acids important for LIYV P26 function and viral infectivity.

**FIG 5:**
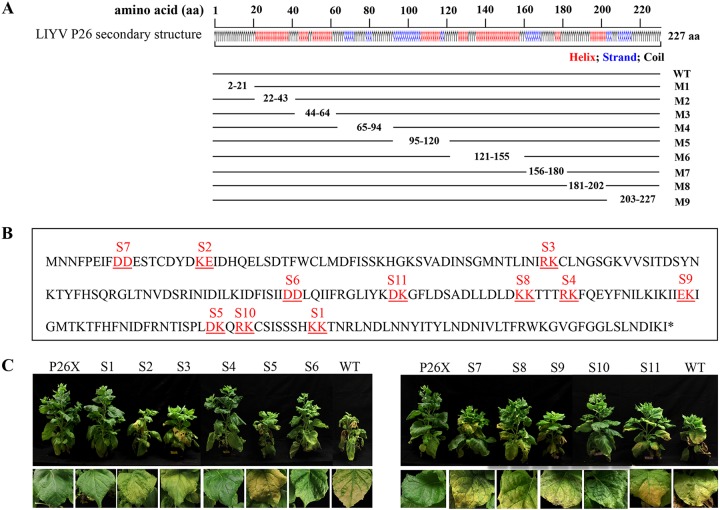
Analysis of structural regions or amino acids important for LIYV P26 function. (A) Schematic representation of nine P26 gene truncation mutants (M1 to M9) generated based on the P26 secondary structure (helix/strand/coil) predicted by the I-TASSER program. (B) Alanine-scanning analysis of the P26 amino acid sequence. Amino acids replaced with alanine are underlined and colored in red, and the names of mutants containing double substitutions are indicated above the mutated residues. (C) Phenotypes of LIYV WT- and P26 mutant-infected N. benthamiana plants photographed at 3 weeks postinoculation.

The alignment of multiple *Criniviru*s P26 ortholog sequences with the LIYV P26 amino acid sequence shows very low similarity (21), and no conserved motifs can be identified from known databases. To locate the regions of LIYV P26 that are critical for *in planta* systemic movement, we took two mutagenesis approaches. First, truncation analysis was performed by generating truncations based on the P26 secondary structure (helix/strand/coil) as predicted by the I-TASSER program. Nine region deletion mutants of P26 (M1 to M9), each comprising truncations of 20 to 35 amino acids, were constructed (Fig. 5A). *In planta* assays of these mutants for virus infectivity showed none of them to be capable of systemic infection, although viral RNA multiplication and LIYV CP expression were detected in their agroinfiltrated leaves, indicating that deletions of any of these regions abolished proper functions of P26 and systemic infection (see Fig. S4A in the supplemental material).

10.1128/mBio.02230-18.4 FIG S4 *In planta* test of LIYV infectivity comprising P26 mutations in Nicotiana benthamiana plants. (A) Virus infectivity and systemic accumulation of LIYV P26 truncation mutants (M1 to M9) tested by RT-PCR and immunoblotting. (Left) Detection of viral RNA accumulation and P26 truncation mutations by RT-PCR with total RNA extracted from upper noninoculated and agroinfiltrated leaves of LIYV WT- and LIYV P26 M1- to M9-agroinoculated plants. An F2 primer set amplifying the sequence flanking the P26 gene was used. RNA samples derived from agroinfiltrated leaves without RT were applied as a negative control. (Right) Subcellular fractionation was applied to concentrate P26 and CP proteins expressed from LIYV WT and LIYV P26 M1 to M9 in agroinoculated leaves for immunoblot analysis. Protein samples extracted from upper leaves of LIYV WT systemically infected plants (WT-U) were used as controls. The Ponceau S-stained RuBisCO large subunit serves as a loading control. P30, 30,000 × *g* pellet; S30, 30,000 × *g* supernatant. (B) Virus infectivity and systemic accumulation of LIYV P26 alanine substitution mutants (S1 to S11) examined by RT-qPCR and immunoblotting. (Left) Quantification of LIYV RNA1 accumulation in upper noninoculated leaves of LIYV WT, P26X, and P26 S1 to S11 mutant-inoculated plants by RT-qPCR. The PP2A transcript level of N. benthamiana was used as an internal control. Error bars denote standard errors from at least three biological replicates. (Right) Immunoblot analysis of LIYV CP accumulation in upper noninoculated leaves using LIYV CP-specific antibody. The Ponceau S-stained RuBisCO large subunit serves as a loading control. Download FIG S4, TIF file, 0.9 MB. Copyright © 2018 Qiao et al. 2018 Qiao et al. This content is distributed under the terms of the Creative Commons Attribution 4.0 International license.

For the second mutagenesis approach, 11 alanine-scanning double-substitution mutants were generated by replacing clustered charged amino acids of P26 with alanine in the LIYV WT infectious clone backbone (Fig. 5B). All 11 mutants were tested for their ability to systemically infect N. benthamiana plants; the LIYV WT and P26X were used as the positive and negative controls. Virus infection in upper leaves of agroinoculated N. benthamiana plants was tested and quantified by real-time quantitative RT-PCR (RT-qPCR) and immunoblotting at 3 wpi. Three mutants (S2, S5, and S9) showed efficient systemic infection with viral accumulation and symptom severity similar to those of the LIYV WT, suggesting these mutations did not affect the proper functions of P26. In contrast, two mutants (S1 and S4) showed no observable symptom development, the same as LIYV P26X, and no LIYV infection was detected from their upper leaves, indicating these regions have a critical role in P26 protein function. All the other mutants (S3, S6, S7, S8, S10, and S11) were capable of systemic infection but showed significantly reduced viral accumulation levels, which was also reflected as milder symptoms compared to those caused by the wild-type LIYV infection (Fig. 5C; Fig. S4B).

The subcellular localization of these mutated P26 proteins was determined by their GFP fusions. All P26 mutants that abolished systemic infection, including M1 to M9, S1, and S4, showed significantly different distribution patterns compared to the wild-type P26 (e.g., P26_M1:GFP relocalized to nucleus, and P26_S1:GFP formed flake-like aggregates floating in the cytoplasm). All others that were still capable of long-distance transport retained the wild-type P26 properties of PD localization and/or aggregation (see Fig. S5 in the supplemental material). Thus, the inability to localize to PD is correlated with the functional incapacitation of these P26 mutants and therefore further supports the indispensable role of P26 in LIYV systemic infection. It is interesting to note that predicted structural analysis of the S1 and S4 mutants did not show obvious differences from WT P26; however, the biological assays here demonstrate that these residues are indeed critical for P26 activity in plants.

10.1128/mBio.02230-18.5 FIG S5 Comparison of subcellular localization of P26:GFP, GFP:P26, P26_M1 to -M9:GFP, and P26_S1 to -S11:GFP in Nicotiana benthamiana epidermal cells at 3 dpi. Scale bars, 10 μm. Download FIG S5, TIF file, 5.7 MB. Copyright © 2018 Qiao et al. 2018 Qiao et al. This content is distributed under the terms of the Creative Commons Attribution 4.0 International license.

## DISCUSSION

Plant viruses initiate their infections from epidermal, mesophyll cells or phloem-specialized cells, depending on virus species and their mode of transmission to the host plant. For long-distance movement, viruses are adapted to move cell to cell by targeting and modifying PDs between different cell types (cellular barriers) until loading into SEs, where they are passively transported with the source-to-sink flow (1). The movement strategies of many plant viruses are increasingly well documented: however, far less is known about viral transport mechanisms of viruses whose infections are phloem limited in their host plants, including those in the families *Closteroviridae* and *Luteoviridae* (2, 26).

Here we showed that the LIYV non-virion protein P26 is a critical determinant allowing LIYV to systemically infect the host plant N. benthamiana. P26 targets PDs in plant cells, is not required for replication or assembly, and appears not to be involved in mitigating RNA silencing (27). The distribution of the wild-type LIYV and the P26X mutant was tracked via GFP expressed from recombinant constructs, and GFP fluorescence was detected over 10 days after inoculation in the agroinfiltrated leaves. Unlike that observed with BYV or CTV, GFP expression was low in epidermal cells, possibly due to the strict phloem limitation of LIYV compared to the others. In contrast, strong GFP fluorescence was detected in the vascular system of plants infected by LIYV-GFP, but not in plants infected by P26X-GFP. These data thus suggest that the P26 null mutant is likely defective in virus loading from initially infected cells into the phloem.

**FIG 6:**
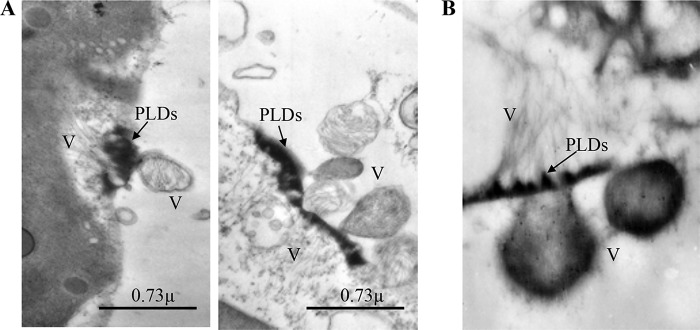
Transmission electron micrographs of LIYV-infected Nicotiana benthamiana protoplasts. (A) Sacks of LIYV virions (V) are external to the plasmalemma directly adjacent to the conical electron-dense plasmalemma deposits (PLDs), and filamentous LIYV virions appear to be oriented perpendicular to PLDs at the internal side. (B) Immunogold labeling using antisera against LIYV CPm.

Unlike other PD-localizing, cytopathology-inducing viral movement proteins, LIYV P26 forms the unique conical electron-dense PLDs that are so far observed only in LIYV-infected host plants (16, 17). PLDs are located over PD pit fields in phloem parenchyma cells and companion cells adjacent to SEs, and virions oriented perpendicular to PM at PLDs are frequently observed (16, 18, 19). Interestingly, our TEM analysis also showed that sacks of LIYV particles were external to the PM of LIYV-infected N. benthamiana protoplasts directly adjacent to the PLDs, confirmed by immunogold labeling with antibodies against the LIYV virion protein CPm (Fig. 6). Thus, at least in protoplasts, LIYV virions can exit the initially infected cells when P26 is present. Taken together, we propose that P26 likely aids the shuttling of virus particles between companion cells and phloem parenchyma cells or phloem loading or unloading of virions into SEs for systemic transport. It is likely that P26 might be interacting with LIYV virion components and/or some host factors, due to the specific association of LIYV particles with the PLDs, to orient virus particles to the cell periphery and/or direct them through the PDs, although when tested by a Y2H assay, P26 was only capable of self-interaction in all possible pairwise combinations with LIYV-encoded proteins (21).

We also showed that the transient expression of P26 via plasmids or from a heterologous TMV vector failed to complement LIYV P26X, although previous work showed that PLDs formed by P26 expressed from TMV were found to be indistinguishable from those produced by LIYV infection (17). However, the movement defect of the P26X was successfully rescued by *cis* expression of a dislocated P26 ORF in the modified LIYV genome. These data suggest that a functional P26 must be expressed not only in cells where LIYV is replicating and virions are assembling, but likely in proper amounts at the proper time and cellular location. Furthermore, there are numerous examples showing that the movement functions are interchangeable between related or even unrelated viruses and that movement-defective phenotypes of certain viruses are complemented by MPs encoded by others (28). TMV MP, for example, has been shown to allow the movement of other, often unrelated viruses (28, 29); however, in our case the systemic infection of LIYV P26X was not observed when coinoculated with recombinant TMV expressing P26. Moreover, P26 orthologs from other criniviruses failed to complement LIYV movement. Whether the orthologs have roles in systemic spread of their respective criniviruses, similar to that shown here for the LIYV-encoded P26, is yet unknown.

LIYV P26 has no similar proteins when compared by database searches, and the P26 amino acid sequences show no significant similarity to other proteins, even orthologs of other criniviruses, nor indication of conserved functional domains or motifs in the existing databases. Our mutational analysis showed that large deletions within P26 abolished is biological activity, but alanine scanning mutation analysis allowed identification of specific residues that are critical for function. Among 11 mutants generated here, 3 still allowed for systemic infection comparable to the wild-type LIYV, while all the others exhibited highly reduced or abolished systemic infection. Interestingly, for the two mutants (S1 and S4) which presented completely disrupted systemic infection, the targeted amino acids are found clustered together on the predicted surface-modeled structure, although substitution for these residues with alanine did not show obvious differences from the predicted structure of WT P26 (see Fig. S6 in the supplemental material). It is possible that these surface-exposed residues are involved in interactions with other virus and/or host factors to facilitate systemic infection in plants.

10.1128/mBio.02230-18.6 FIG S6 Surface model of P26 generated using the UCSF Chimera program based on the secondary structure predicted by I-TASSER. P26 alanine substitution mutation sites are colored and labeled. Red, S1 and S4 P26 mutants abolished LIYV systemic infection; yellow, S2, S5, and S9 P26 mutants showed efficient systemic infection comparable to the wild type; green, S3, S6, S7, S8, S10, and S11 P26 mutants caused significantly reduced viral accumulation. Download FIG S6, TIF file, 1.6 MB. Copyright © 2018 Qiao et al. 2018 Qiao et al. This content is distributed under the terms of the Creative Commons Attribution 4.0 International license.

## MATERIALS AND METHODS

### Plasmid construction and agroinfiltration.

All plasmid constructs used in this work were generated using In-Fusion HD cloning (Clontech) following the manufacturer’s instructions. For transient and heterologous expression of P26, the P26 gene was cloned and assembled into the pEAQ-HT and TMV pJL24 vectors (25, 30). P26:GFP and GFP:P26 were generated by introducing the GFP ORF into the N- or C-terminal regions of the P26 ORF in pEAQHT-P26. For P26 mutants, the mutagenesis was introduced into the existing constructs using primers incorporating the mutations or outside the unwanted region and then recircularized into desired clones. To modify LIYV RNA2 WT and P26X for the inclusion of an independent gene expression cassette for coexpression of the LIYV P26 protein, the P26 expression cassette in the pEAQHT-P26 vector was amplified and integrated next to the expression cassette of LIYV RNA2 WT or P26X in the plasmids, generating the WT-35SP26 and P26X-35SP26 clones. The P26X-GFP and P26X-P26 constructs were modified from the previously developed GFP-tagged LIYV RNA2 clone by introducing the same stop codons of P26X into the P26 ORF and replacing the GFP sequence with P26 (24). P26 homolog gene sequences from BPYV, CYSDV, LCV, and ToCV were cloned and ligated into the linearized plasmid of the LIYV RNA2 clone without the LIYV P26 ORF. All clones were verified by DNA sequencing prior to further analysis. Plasmid constructs were delivered to fully expanded leaves of the HC-Pro transgenic N. benthamiana plants at the 4- to 6-leaf stage for Agrobacterium tumefaciens (strain GV3101)-mediated transient expression or virus transfection as described before (20).

### Fluorescence microscopy.

The subcellular localization of the P26-GFP fusion proteins within agroinfiltrated N. benthamiana epidermal cells was examined using a Leica TCS SP2 inverted confocal microscope (Leica Microsystems) under a 63× water immersion objective. The GFP expression of WT-GFP and P26X-GFP in N. benthamiana leaves was observed using a Leica DM5000 B fluorescence microscope (Leica Microsystems); the epidermal layer was removed from the lower side of leaf tissue for a clearer view of the GFP fluorescence in the vascular systems. GFP distribution in the stems and roots of N. benthamiana plants was visualized directly with a long-wavelength UV light and photographed using a Canon EOS 600D digital camera.

### RT-PCR and RT-qPCR.

LIYV infection was detected by RT-PCR and RT-qPCR at the time points indicated. Total RNAs were extracted from agroinfiltrated or newly emerging leaves using the TRIzol reagent (Invitrogen) and treated with DNase (Qiagen) following the manufacturer’s instructions. LIYV RNA accumulation and/or P26 mutations were checked by RT-PCR, the first-strand cDNAs of the negative strand were generated by reverse transcription reactions with forward primers LIYV-CP_F and LIYV-P26_F and SuperScript II reverse transcriptase (Invitrogen), and PCR was carried out to amplify the partial LIYV CP-coding region using primer set F1 and sequence covering the LIYV P26-coding region using primer set F2. For the P26X-P26 construct, the integrity of the original P26 ORF containing the knockout mutations and the translocated P26 ORF was checked by RT-PCR with primer sets F3 and F4. The systemic infection of TMV-GFP and TMV-P26 constructs was examined by RT-PCR with TMV-IN primers flanking the insertions. The viral RNA accumulation level was quantified by RT-qPCR as described before (24). The primer sequences mentioned above are listed in Table S1 in the supplemental material.

10.1128/mBio.02230-18.7 TABLE S1 Primer sequences used for RT-PCR and RT-qPCR. Download Table S1, DOCX file, 0.01 MB. Copyright © 2018 Qiao et al. 2018 Qiao et al. This content is distributed under the terms of the Creative Commons Attribution 4.0 International license.

### Immunoblot analysis.

Total protein extraction, LIYV virion isolation, and protein subcellular fraction were performed as previously described (31–33). The protein samples prepared were analyzed by SDS-PAGE and immunoblotting following the protocol described before (16). Primary antibodies used included anti-GFP (Thermo), anti-LIYV CP, anti-LIYV P26, and anti-LIYV virion protein polyclonal antibodies produced in rabbits. The secondary antibody was horseradish peroxidase-conjugated goat anti-rabbit IgG.
