# Supplementary figures and images for: A Distinct, Non-Virion Plant Virus Movement Protein Encoded by a Crinivirus Essential for Systemic Infection
Source: mBio. 2018 Nov 20;9(6):e02230-18. doi: 10.1128/mBio.02230-18 (PMC6247084; doi:10.1128/mBio.02230-18)

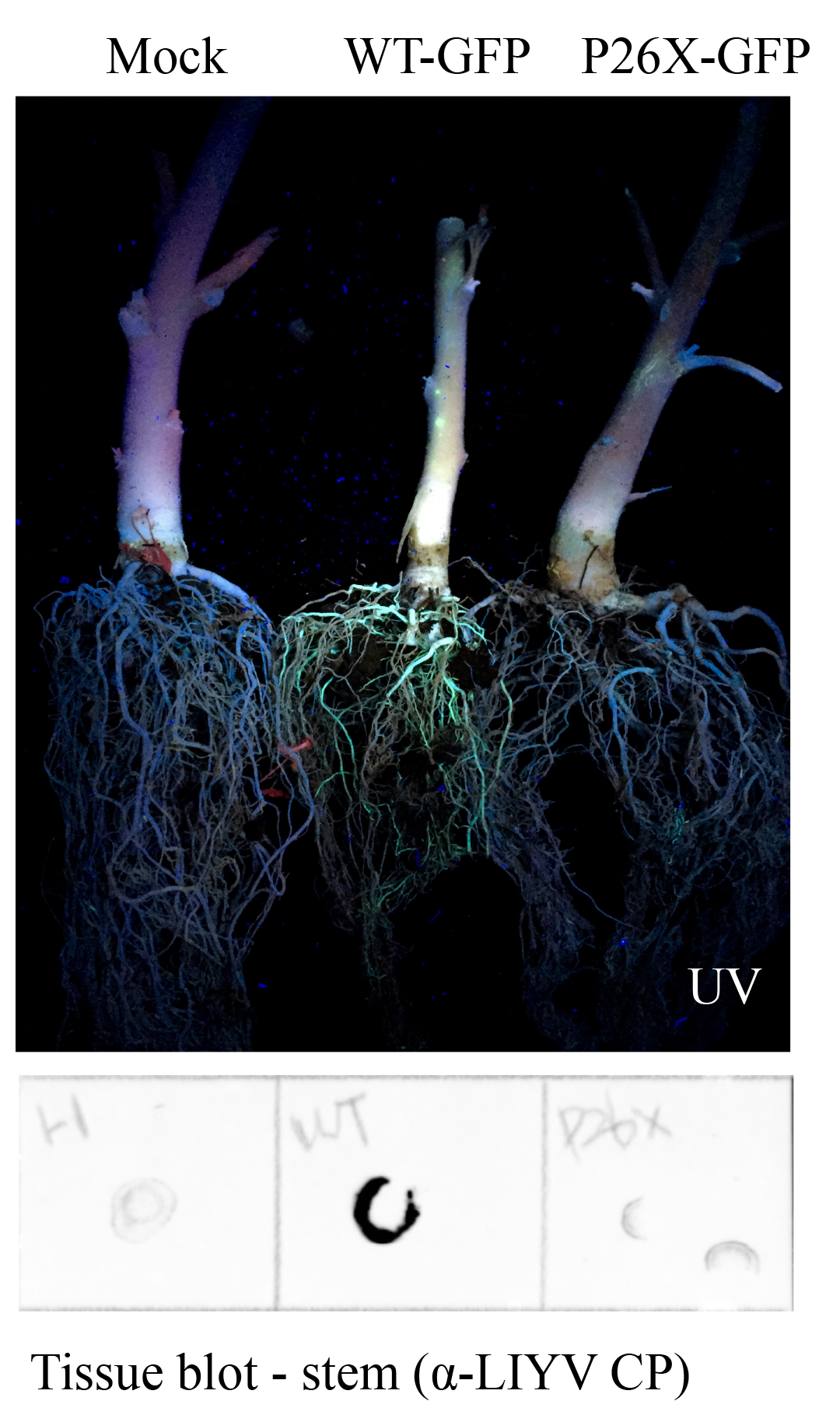

Supplement: FIG S1 [file mbo006184182sf1.tif]

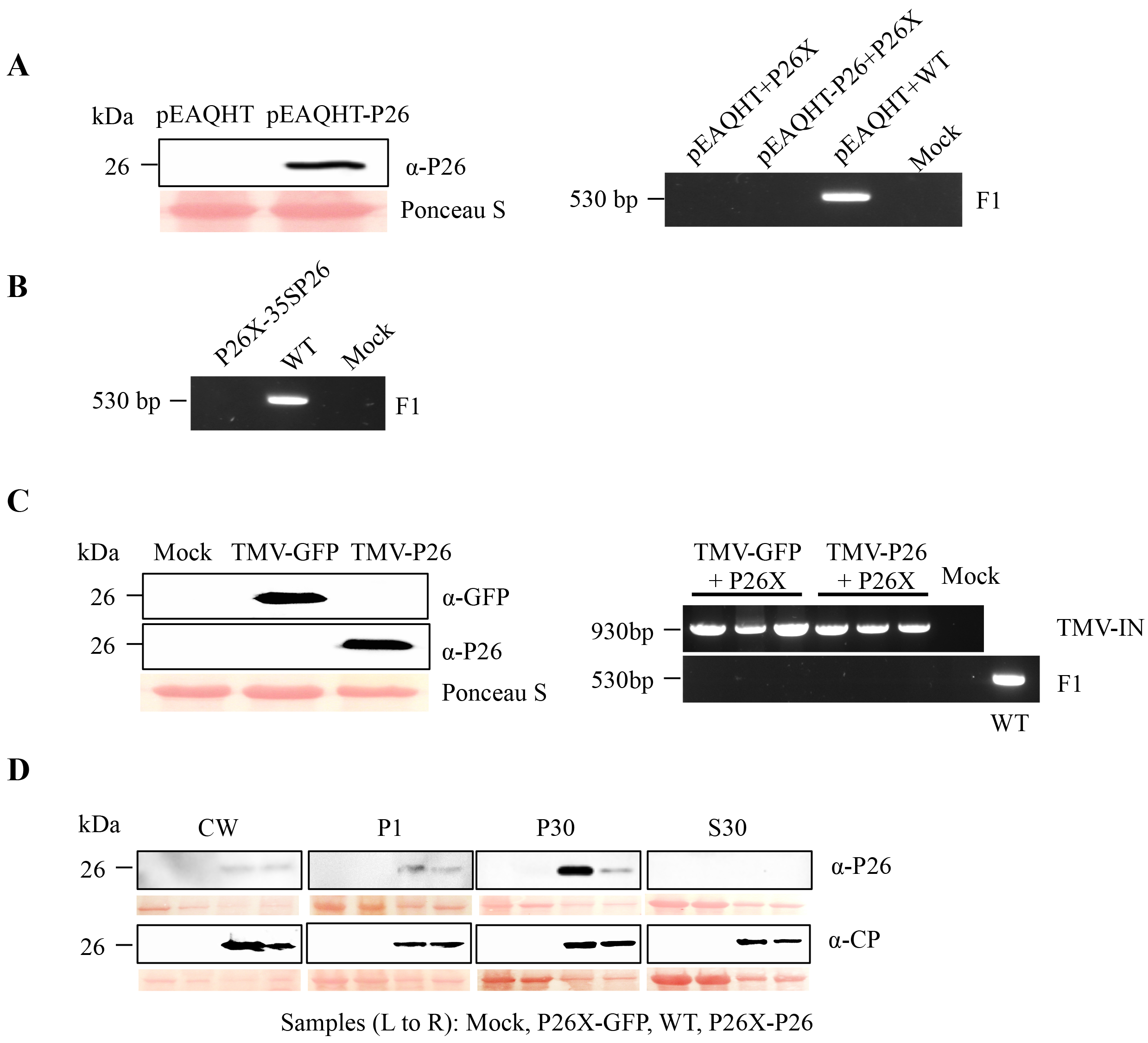

Supplement: FIG S2 [file mbo006184182sf2.tif]

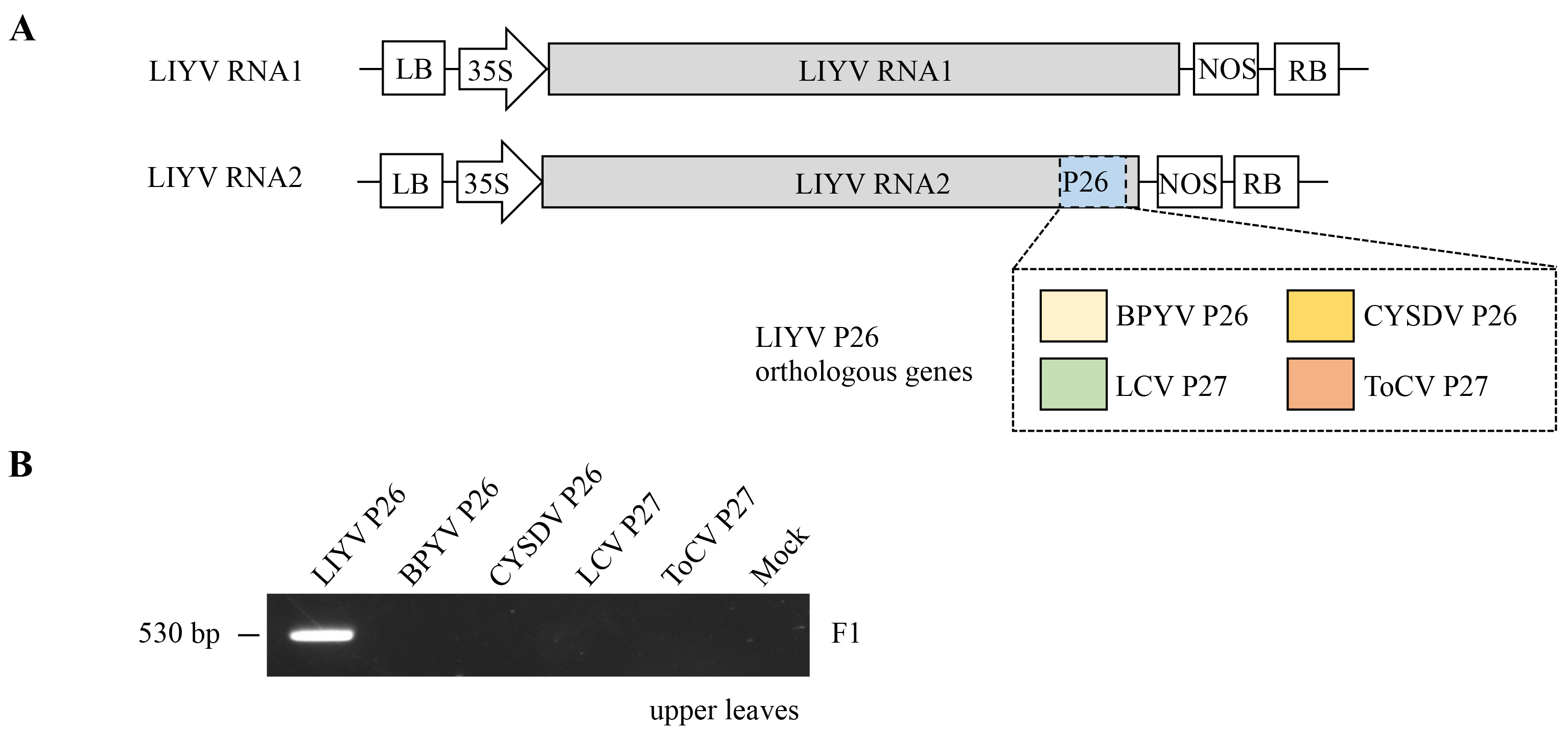

Supplement: FIG S3 [file mbo006184182sf3.tif]

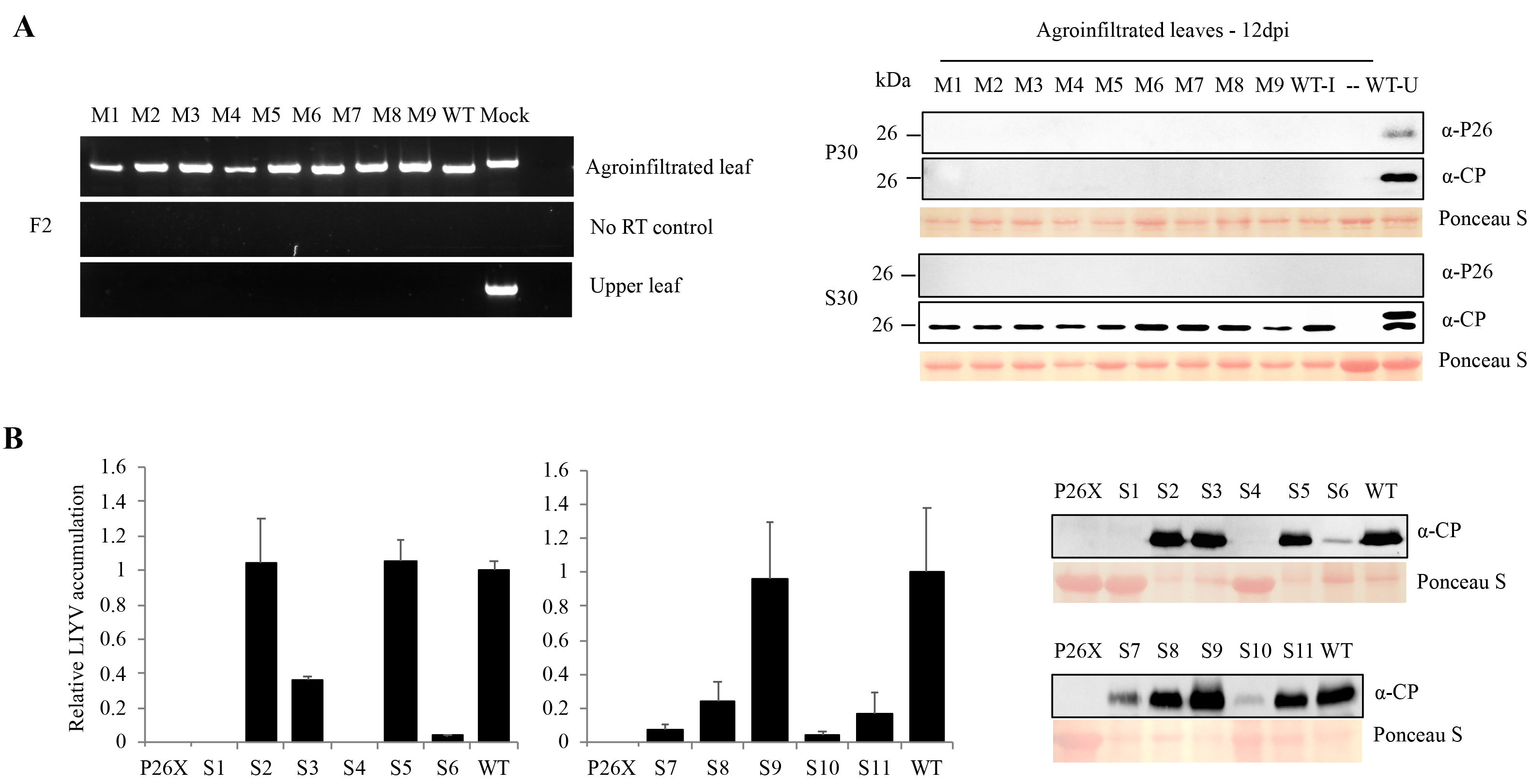

Supplement: FIG S4 [file mbo006184182sf4.tif]

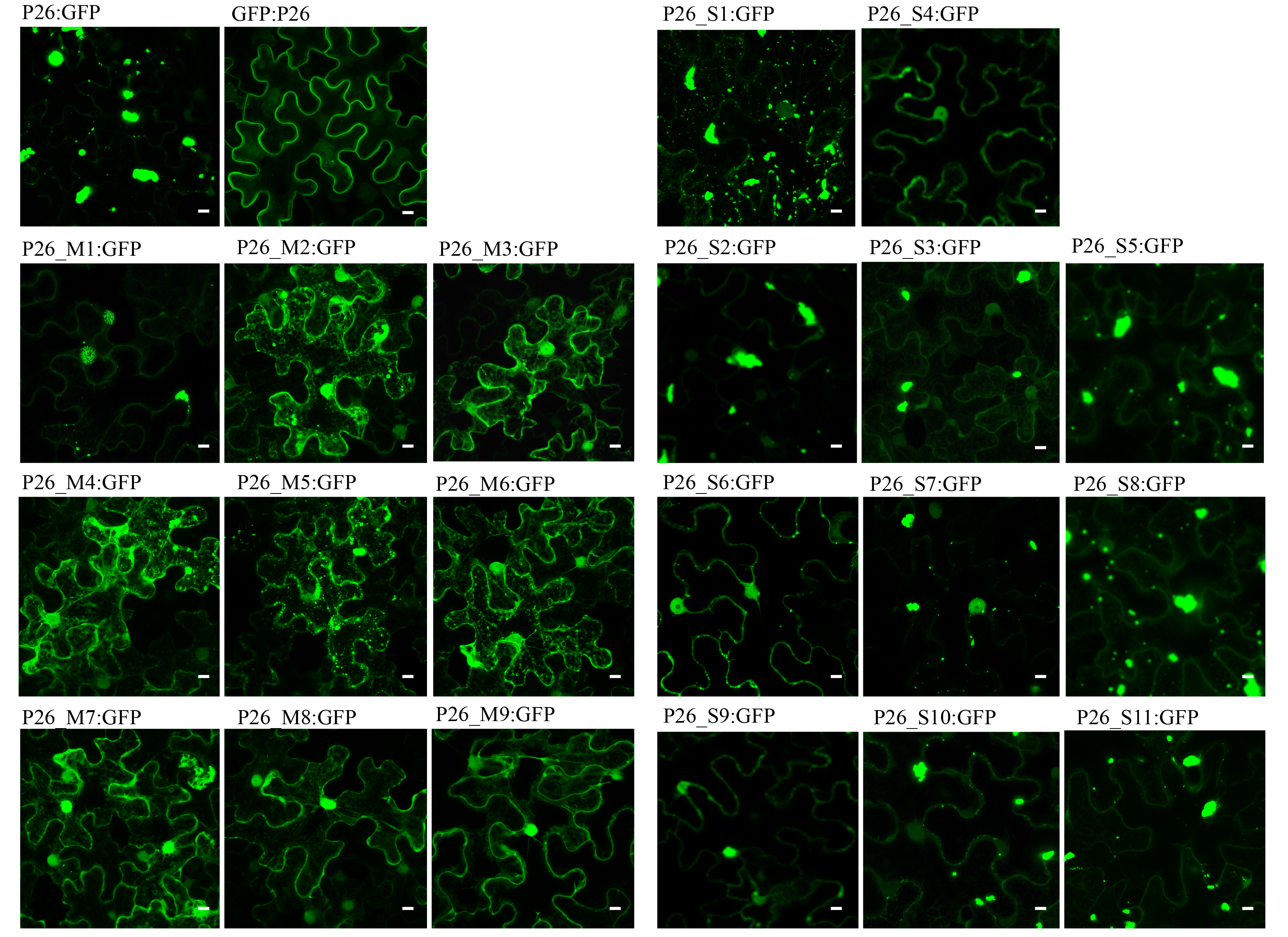

Supplement: FIG S5 [file mbo006184182sf5.tif]

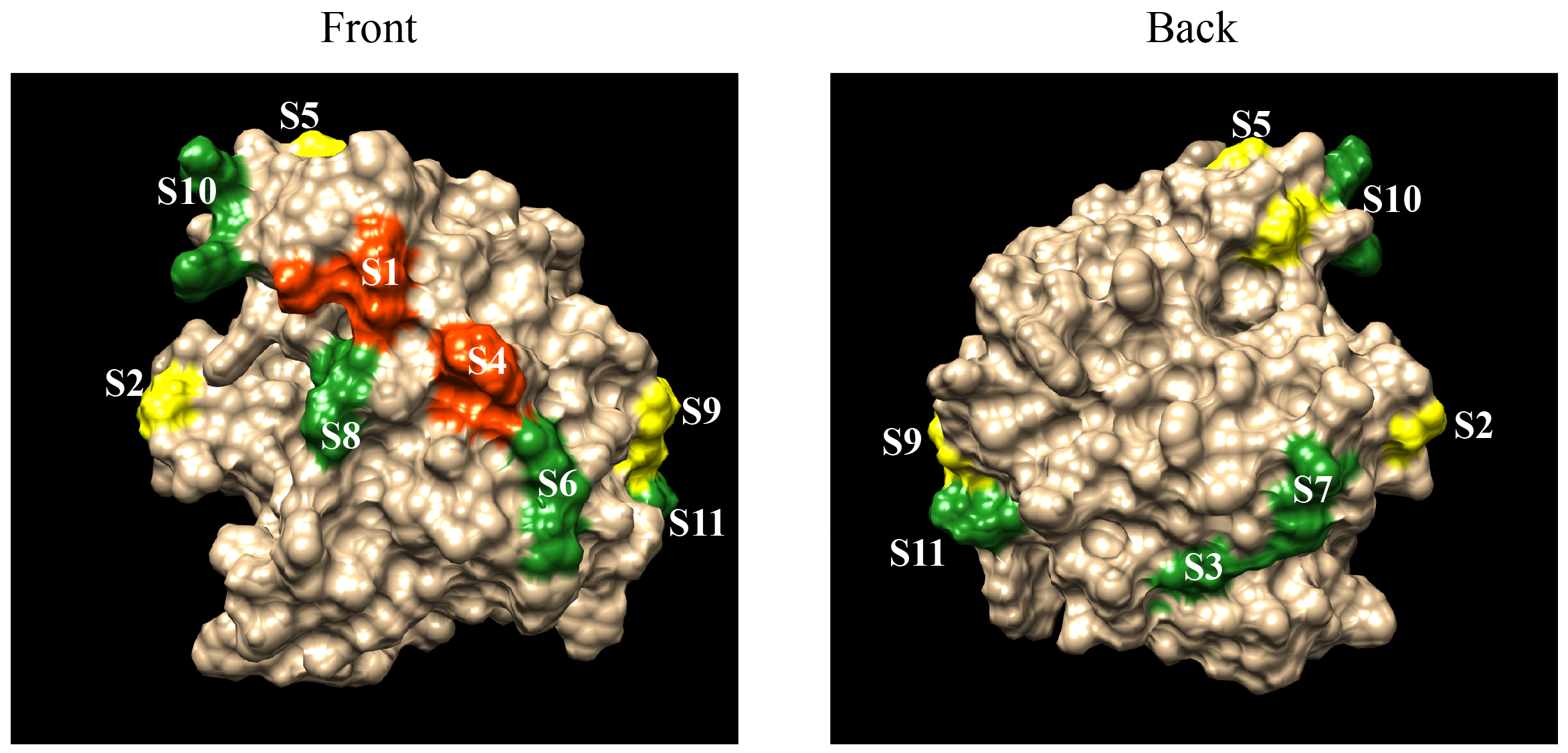

Supplement: FIG S6 [file mbo006184182sf6.tif]
