# Supplementary material for: A Distinct, Non-Virion Plant Virus Movement Protein Encoded by a Crinivirus Essential for Systemic Infection
Source: mBio. 2018 Nov 20;9(6):e02230-18. doi: 10.1128/mBio.02230-18 (PMC6247084; doi:10.1128/mBio.02230-18)
Supplement: TABLE S1 [file mbo006184182st1.docx]

| **Primer sets** | **Primer sequences** |
| --- | --- |
| **For RT-PCR** | |
| F1 | LIYV-CP_F:  5’-TTGTCCAAAATCGTTGTACGCG-3’  LIYV-CP_R:  5’-CCACCTTCACCTTTGCATAATG-3’ |
| F2 | LIYV-P26_F :  5’-GTGTTAGAATGGCCTAGGAAATTAGC-3’  LIYV-P26_R:  5’-ATCGAATAATTCAATCACCACTCTCTGATCA-3’ |
| F3 | LIYV-P26_F:  5’-GTGTTAGAATGGCCTAGGAAATTAGC-3’  F3_R:  5’-TGAATCTAAACAGAACTCAAGGAAATCTAGG-3’ |
| F4 | F4_F:  5’-TTGAGGTTTCTAGTCAATTTATTAAAAACTACAG-3’  F4_R:  5’-TTAAATTTTTATATCATTTAATGATAGTCCTCCGAATCCTACTCC-3’ |
| TMV-IN | F:  5’-TCGATGATGATTCGGAGGCTA-3’  R:  5’-CATATACATTTGACCCGCGCGATC-3’ |
| **For RT-qPCR** | |
| LIYV RNA1 | F:  5’-TGTTCGCCCAGGT-TAGATTTG-3’  R:  5’-TTCACCATATCCTTTCAGCCC-3’ |
|  | Probe: 5′-AGACACATCCAAAGGGCCACAGT-3′ |
| *N. benthamiana*  PP2A | F:  5’-GAGAAAACCATTCGCCCTAGT-3’  R:  5’-GACTGAAGTGCTTGATTGGC-3’ |
|  | Probe: 5′-CTGAAGACCCTGATGTTGATGTTCGCT-3′ |
